# Left ventricular function: time-varying elastance and left ventricular aortic coupling

**DOI:** 10.1186/s13054-016-1439-6

**Published:** 2016-09-10

**Authors:** Keith R. Walley

**Affiliations:** Centre for Heart Lung Innovation, St. Paul’s Hospital, University of British Columbia, 1081 Burrard Street, Vancouver, BC V6Z 1Y6 Canada

## Abstract

Many aspects of left ventricular function are explained by considering ventricular pressure–volume characteristics. Contractility is best measured by the slope, Emax, of the end-systolic pressure–volume relationship. Ventricular systole is usefully characterized by a time-varying elastance (ΔP/ΔV). An extended area, the pressure–volume area, subtended by the ventricular pressure–volume loop (useful mechanical work) and the ESPVR (energy expended without mechanical work), is linearly related to myocardial oxygen consumption per beat. For energetically efficient systolic ejection ventricular elastance should be, and is, matched to aortic elastance. Without matching, the fraction of energy expended without mechanical work increases and energy is lost during ejection across the aortic valve. Ventricular function curves, derived from ventricular pressure–volume characteristics, interact with venous return curves to regulate cardiac output. Thus, consideration of ventricular pressure–volume relationships highlight features that allow the heart to efficiently respond to any demand for cardiac output and oxygen delivery.

## Background

The heart is a muscle pump that delivers blood at a high pressure to drive passive blood flow through a complex arterial and venous circuit. The demand for blood flow is determined by metabolic activity of the tissues. For example, increased skeletal muscle work leads to increased demand for oxygen so blood flow must increase to this specific muscle. Since the need for blood flow is determined by peripheral tissue demands, it follows that blood flow must be regulated by the periphery. So the heart must have special characteristics that allow it to respond appropriately and deliver necessary blood flow and oxygen, even though flow is regulated from outside the heart.

To understand these special cardiac characteristics we start with ventricular function curves and show how these curves are generated by underlying ventricular pressure–volume characteristics. Understanding ventricular function from a pressure–volume perspective leads to consideration of concepts such as time-varying ventricular elastance and the connection between the work of the heart during a cardiac cycle and myocardial oxygen consumption. Connection of the heart to the arterial circulation is then considered. Diastole and the connection of the heart to the venous circulation is considered in an abbreviated form as these relationships, which define how cardiac output is regulated, stretch the scope of this review. Finally, the clinical relevance of this understanding is highlighted by considering, for example, why afterload reduction is an excellent therapy for systolic heart failure but fails to help, and instead harms, when systolic heart failure is not the problem.

## Ventricular function curves

### Classic ventricular function curves

**Fig. 1:**
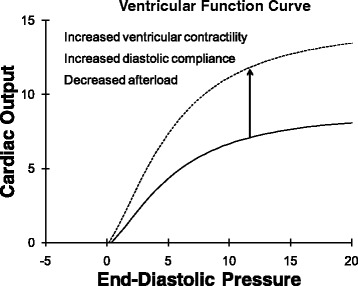
This classic ventricular function curve relates input of the heart (end-diastolic pressure in mmHg) to output of the heart (cardiac output in liters per minute). The ventricular function curve shifts up and to the left when ventricular systolic contractility increases. However, increased diastolic compliance and decreased afterload can also shift the ventricular function curve up and to the left

Starling function curves, or ventricular function curves, relate the input of the heart to output (Fig. 1) [1]. Any input and any output can be considered. For the heart we most frequently use right atrial pressure (central venous pressure) or left atrial pressure (pulmonary capillary wedge pressure) as clinically measurable inputs to the heart (preload). Cardiac output (blood flow out of the heart in liters per minute) is a common measure of output of the heart. Other inputs and outputs yield different but related ventricular function curves.

As the preload of the heart increases, cardiac output increases—sometimes called Starling’s law of the heart or the Frank–Starling relationship [2]. This relationship is curvilinear (Fig. 1) so that, at high preload, further increases in preload yield diminishing increases in cardiac output. A specific ventricular function curve applies to a specific ventricular contractile state, diastolic compliance, and afterload. Using cardiac output as pump output, if ventricular contractility increases (at a constant afterload), then the ventricular function curve shifts up and to the left so that, at the same end-diastolic pressure (preload), a greater stroke volume and cardiac output is achieved [1] (Fig. 1, dashed line). Improved ventricular diastolic compliance and decreased afterload (aortic pressure) also results in increased stroke volume (at a constant contractile state) so these changes also shift the ventricular function curve up.

### Modified ventricular function curves

The motivation behind early studies of ventricular function curves was the desire to identify intrinsic ventricular contractile function. Classic Starling function curves shift down with increased afterload [3] without a change in contractility of the heart [4]. Therefore, modifications to ventricular function curves were considered. In some versions “output” was changed to stroke work or stroke work per minute (stroke power) [5]. Stroke work incorporates afterload since it is pressure afterload × stroke volume. In some versions “input” was changed to end-diastolic volume [6]. This addressed the issue of non-linear diastolic compliance [5]. Probably the zenith of modifications to ventricular function curves is the concept of preload recruitable stroke work [7]. When stroke work is plotted against end-diastolic volume the relationship is highly linear and fairly insensitive to loading conditions. The slope of this relationship is a measure of ventricular contractility [7]. These modified ventricular function curves are more specific for intrinsic ventricular contractile function but fall short of perfect. Because of different strengths and weaknesses, different variants of ventricular pump function inputs and outputs can be chosen strategically to address specific questions. In a clinical context central venous pressure (right ventricular end-diastolic pressure) and cardiac output are readily measurable and are often most appropriate.

Alternative approaches to measurement on intrinsic myocardial contractility included consideration of the rate of change of ventricular chamber pressure during isovolumic systole—before “afterload” is seen by the contracting ventricle. The maximum rate of change of ventricular pressure, dP/dt_max_, occurs late in isovolumic systole and increases when contractility is increased [8] (for example, by addition of adrenergic agents or calcium). While dP/dt_max_ avoids the problem of afterload effect, dP/dt_max_ is sensitive to changes in preload. Not surprisingly, when isovolumic contraction starts at a greater end-diastolic volume (V_ED_) then dP/dt_max_ is greater. Therefore, empirical corrections have been applied. (dP/dt_max_)/V_ED_ is another adjusted measure of ventricular contractile function [9]. An interesting extension relates these isovolumic pressure measurements to sarcomere shortening. Vmax [6], the maximum rate of sarcomere shortening, was calculated by considering units of cardiac muscle (sarcomeres) to be made up of a contractile element and a linear series elastic element—the series elastic element converting contractile element shortening into pressure. In analogy to cardiac muscle velocity–length relationships [10], the maximum velocity of shortening can be extrapolated from a plot of dP/dt (representing velocity of contractile element shortening) versus ventricular pressure (representing contractile element length). This approach appeared to circumvent afterload sensitivity and incorporated preload. However, these and related computed indices all remain sensitive to changes in preload, afterload, and heart rate to varying degrees.

“Curve-fitting” approaches to modifying ventricular function curves did not conceptually connect easily with underlying mechanism—Hill’s sliding filament model of muscle contraction. While isovolumic phase measurements were linked to underlying mechanism, they required many debatable assumptions. Consideration of ventricular pressure–volume relationships made the connection to underlying mechanism and incorporated the concepts of preload, afterload, diastolic compliance, and contractility [11].

## Ventricular pressure–volume relationships

### Underlying muscle force–velocity and force–length relationships

**Fig. 2:**
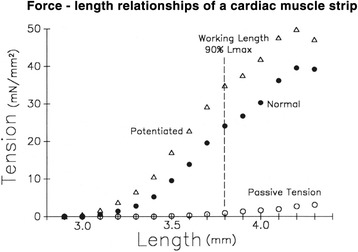
For an isolated rabbit trabecular muscle strip, force, expressed as force per area = tension, is plotted against starting length. Passive tension during diastole is plotted as *open circles*. Normal active tension after electrical stimulation is plotted as *closed circles*. Contractions following a second rapid electrical stimulation, which increases the calcium concentration at actin/myosin sliding filaments and therefore increases contractility (*Potentiated*), is plotted as *open triangles*

Cardiac muscle strips demonstrate length-dependence of systolic contractile force (Fig. 2). As the strip of relaxed cardiac muscle is stretched, passive tension rises, but not much. That is, the relaxed diastolic muscle strip is very compliant. When a contraction is elicited by an electrical stimulation the muscle becomes much stiffer so tension (force per area) rises to a maximum systolic value. When the contraction occurs at a greater initial length then maximum systolic force increases substantially—an expression of Starling’s law of the heart. This force–length relationship is quite linear but, at extreme muscle lengths, the relationship plateaus at a maximum tension in part because, with increasing stretch, overlap between actin and myosin filaments reaches a maximum and then decreases. Maximum overlap corresponds to a maximum number of actin–myosin cross-bridges and, hence, maximum force. This force–length characteristic curve describes inherent cardiac muscle properties at one contractile state.

Repeat stimulation of the cardiac muscle strip shortly after the initial stimulation (potentiated contraction) causes release of further calcium from the sarcoplasmic reticulum into the sarcoplasm, causing increased interaction of actin and myosin to result in increased contractility (Fig. 2). The slope of the potentiated force–length relationship increases. Thus, an increase in the inherent contractility of a cardiac muscle strip results in a shift up and to the left of the force–length relationship, primarily characterized by an increase in slope.

Cardiac muscle strips arranged into a three-dimensional, somewhat spherical structure, the heart, then generate pressure due to muscle strip force, at a ventricular chamber volume that relates to underlying muscle strip length. Thus, cardiac muscle force–length relationships underlie ventricular pressure–volume relationships and, therefore, share a number of key features [12].

### Ventricular pressure–volume loops

**Fig. 3:**
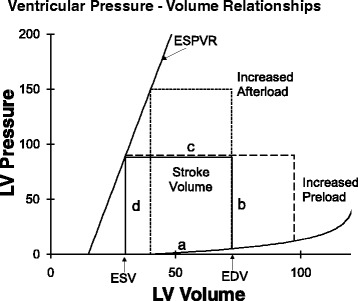
Left ventricular pressure volume relationships. A cardiac cycle is illustrated by the loop labeled as “a”, “b”, “c”, and “d”. *ESPVR* end-systolic pressure–volume relationship, *LV* left ventricular

To remove and control the influence of changes in preload and afterload, several groups of investigators studied isolated perfused hearts with loading conditions controlled using servo systems. Starling’s very early work demonstrated that increasing end-diastolic pressure and volume increased stroke volume. The effect of afterload was considered next. Weber, Janicki, and colleagues found that stroke volume decreased linearly with increasing end-systolic pressure [4]. Suga, Sagawa, and colleagues put these concepts together within a ventricular pressure–volume diagram (Fig. 3) which illustrates the pressure–volume trajectory of the left ventricle throughout the cardiac cycle and, in particular, illustrates the effect of altered preload and altered afterload [13]. The key feature is that, at the same contractile state, all contractions end on the same end-systolic pressure–volume relationship (ESPVR).

Figure 3 illustrates that during diastole the heart fills at quite low pressures along the normally compliant diastolic pressure–volume relationship of the ventricle (labeled “a”). With the onset of isovolumic systole the ventricle contracts, raising intraventricular pressure at constant volume (in the absence of regurgitant valvular heart disease; labeled “b”). When ventricular pressure exceeds aortic pressure the aortic valve opens and ejection occurs (labeled “c”) and continues to an end-systolic pressure–volume point that lies on the ESPVR. Intraventricular pressure decreases during the isovolumic relaxation phase (labeled “d”) and the cardiac cycle starts again.

#### Diastolic filling “a”

The diastolic pressure–volume relationship is highly compliant so that the ventricle fills easily at low diastolic filling pressures. The relationship is curvilinear, fitted well with an exponential relationship [14] so that at increasing volumes the ventricle becomes increasingly stiff. This curvilinear diastolic pressure–volume relationship is also fit well with a mathematically similar relationship, P = S × log[(Vm − V)/(Vm − Vo)], where S represents diastolic myocardial stiffness, Vm is a maximum diastolic ventricular volume (set by the pericardium and the cardiac cytoskeleton [15, 16]), and Vo is diastolic volume at a pressure of zero [17]. This relationship is asymptotic to the maximum diastolic volume, Vm, so that maximum volume is reached even if pressure continues to rise; a feature which likely better approximates the biological reality that a ventricle can not expand indefinitely [18]. Maximum volume is set by the maximum dimensions of the heart [19] and pericardium [20]. Above the maximum volume the ventricle would rupture. Either equation conveys the key characteristic of the diastolic ventricle—it is highly compliant at low volumes and fills easily but becomes much stiffer as it approaches a maximum diastolic volume.

#### Isovolumic contraction “b”

Systole starts with active contraction of ventricular muscle. This increase in ventricular wall tension is translated into an increase in intraventricular pressure via the LaPlace relationship. That is, for an approximately spherical ventricle Intraventricular pressure = (Wall tension × Wall thickness × 2)/Radius. In normal hearts, this rise in intraventricular pressure closes the mitral valve. Since intraventricular pressure is less than aortic pressure, the aortic valve is also closed. Therefore, during this phase of ventricular contraction, there is no change in ventricular volume. The rate of rise of pressure during isovolumic systole has been used as a measure of intrinsic ventricular contractile function. In particular, the maximum rate of rise of intraventricular pressure, dP/dt_max_, (dP/dt_max_)/V_ED_, and Vmax are frequently used [21].

#### Ejection phase “c”

As systolic ventricular contraction continues intraventricular pressure rises and then exceeds aortic pressure, which opens the aortic valve and ejection of blood occurs. Ejection continues until end-systole. Stroke volume (SV) is the volume of blood ejected with each cardiac cycle and equals end-diastolic volume minus end-systolic volume (SV = V_ED_ − V_ES_). Stroke volume is linearly dependent on afterload and, specifically, on end-systolic pressure.

It is not surprising that at high afterload (the blood pressure along segment “c”) the ventricle is not able to eject far whereas at lower afterload the ventricle is able to eject further. The remarkable finding by Suga and Sagawa and others is that end-systolic pressure–volume points for differently loaded ejections all fall along an approximately linear end-systolic pressure–volume relationship (ESPVR in Fig. 3). That is, an increase or decrease in afterload results in a linearly related decrease or increase, respectively, in ventricular ejection [4] so that the end-systolic pressure–volume point lies on the same ESPVR [13]. Furthermore, if preload is increased (or decreased) so that end-diastolic volume is increased (or decreased) the subsequent stroke volume is increased (or decreased) to the same extent so that the end-systolic pressure–volume point still lies on the same ESPVR [22].

### The end-systolic pressure–volume relationship and Emax

The ESPVR is approximately a straight line with slope Emax. The units of this slope are ΔP/ΔV, which is “elastance”. (Note that the inverse of elastance is compliance, ΔV/ΔP.) The ESPVR incorporates afterload so that indices of ventricular contractility derived from the ESPVR are independent of afterload [23]. Emax is an excellent measure of intrinsic ventricular contractility, which is less load sensitive than other indices of ventricular contractility [6] and is insensitive to heart rate within the normal physiologic range [23]. If Emax increases, it can be seen (Fig. 3) that the ventricle is able to eject further (to a smaller end-systolic volume) at the same afterload, i.e., it demonstrates increased contractility [22].

### Time-varying elastance

**Fig. 4:**
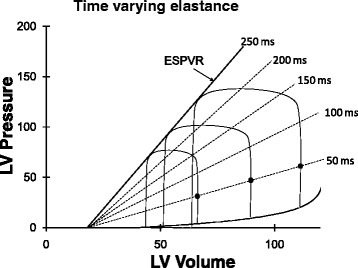
Three differently loaded cardiac cycles. The end-systolic pressure–volume points all lie on a line termed the end-systolic pressure–volume relationship (*ESPVR*). The slope of the ESPVR is Emax, maximum elastance. At any time during systolic contraction (e.g., 50-ms time points are shown as *filled circles*) a line can be drawn connecting pressure–volume points from each of the differently loaded contractions defining elastance (ΔP/ΔV) at that time point. Ventricular systolic contraction can therefore be regarded as a time-varying elastance

The ESPVR is the pressure–volume characteristic curve of the ventricle at end-systole. In an experimental setting the ESPVR is typically determined by measuring the end-systolic pressure–volume points for several differently loaded cardiac cycles to yield several points along a linear relationship—the ESPVR (Fig. 4) [22]. It is also possible to construct pressure–volume characteristic curves for other time points during the cardiac cycle. For example, pressure–volume points at 50 milliseconds into the cardiac cycle can just as easily be measured (Fig. 4). Since the slope of each of these lines is ΔP/ΔV (elastance at the specific time point), the cardiac cycle can be regarded as cyclical changes in elastance of the ventricular chamber—time varying elastance [24]. Thus, the muscular ventricular chamber simply cycles from low elastance (high compliance—diastole) to a high elastance (low compliance—end-systole) state. The presence of mitral and aortic valves cause the time-varying elastance ventricular chamber to describe a pressure–volume loop. Maximum elastance, Emax, occurs very near the end of systole. Because of inertia of blood exiting the aortic valve and aortic impedance, blood flow persists just slightly beyond the time of maximum elastance so Emax does not exactly equal ventricular elastance at end-systole, Ees, but these two measures are nearly equal. The time course of elastance through the cardiac cycle does not change substantially with changes in heart rate [23] but changes markedly with changes in contractility and, indeed by definition, is the full representation of contractility.

During diastole ventricular elastance is at a minimum or, said another way, ventricular compliance is at a maximum, to facilitate rapid filling of the ventricle at low pressures. The diastolic pressure–volume relationship is not exactly linear since the diastolic ventricle becomes stiffer (less compliant, more elastant) as it nears its intrinsic maximum volume and as it impinges upon the constraint provided by the much stiffer pericardial sac. This points out that pressure–volume characteristic curves at any time point during the cardiac cycle are not completely linear from their diastolic pressure–volume relationship start through to their end-systolic pressure–volume relationship maximum [25], but it is impressive that they are nearly so.

### Other features of pressure–volume relationships

The ESPVR is not completely linear because the systolic ventricle has a limit to the maximum pressure that it can generate [25]. Thus, at high volumes and pressures the end-systolic pressure–volume point falls below an exactly linear ESPVR. When the ventricle ejects quickly the end-systolic pressure–volume point falls slightly below an ESPVR generated from slower ejections (or from isovolumic contractions). This is due to internal myocardial viscoelastance. That is, energy is used to overcome viscoelastant characteristics of myocardial muscle.

One perspective is that pressure–volume loops of a cardiac cycle are constrained by the diastolic pressure–volume relationship below and the ESPVR above. Thus, a shift up of the diastolic pressure–volume relationship (decreased diastolic compliance) or a shift down of the ESPVR reduces the available operating space for the heart and, ultimately, leads to heart failure. Another perspective of the cardiac cycle illustrated by pressure–volume loops is that the amount of mechanical work performed by the ventricle during a cardiac cycle is the integral of pressure and volume, which is simply the area of the interior of the pressure–volume loop.

## Pressure–volume relates to metabolic and mechanical function

### Pressure–volume area and myocardial oxygen consumption

**Fig. 5:**
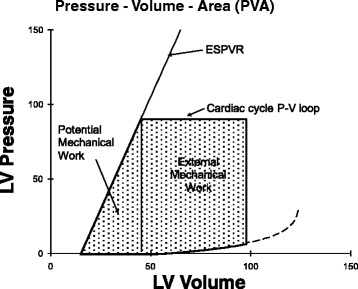
Pressure–volume area (PVA) is the area within the cardiac cycle pressure–volume (*P-V*) loop plus the area under the ESPVR. PVA linearly correlates with myocardial oxygen consumption. *LV* left ventricular

Work that the ventricle performs must be related to the amount of energy consumed as reflected by myocardial oxygen consumption. However, careful measurement shows the area within a pressure–volume loop (stroke work) is not uniformly related to myocardial oxygen consumption per beat [26]. For example, consider an isovolumic contraction and relaxation with no ejection. There is no area within this vertical line on a ventricular pressure–volume diagram yet the ventricle certainly consumes oxygen [26]. Suga and colleagues considered that the additional area underneath the ESPVR could be regarded as potential mechanical work [6] (Fig. 5). The sum of the area within the cardiac cycle pressure–volume loop plus the additional area under the ESPVR was called the pressure–volume area (PVA) [6]. Now the relationship between myocardial oxygen consumption and PVA is linear with a slope of ~30 %, which reflects the efficiency of the heart in converting chemical energy into mechanical energy [27]. This line intersects the myocardial oxygen consumption axis so that, even when producing no PVA work, the heart must consume oxygen to maintain basal cellular function and to recycle calcium back into the sarcoplasmic reticulum with each beat (termed E–C coupling) [28].

An increase in heart rate did not alter the relationship between PVA and myocardial oxygen consumption per beat [29]. However, an increase in contractility, induced by increased calcium or by epinephrine, results in a parallel shift of the relationship [28]. The slope stays the same, which means that an increment in the chemical energy required to produce an increment in PVA work stays the same. However, the myocardial oxygen consumption intercept increases, indicating that the heart is consuming more energy cycling a greater flux of calcium back into the sarcoplasmic reticulum with each beat.

An alternative perspective is that myocardial oxygen consumption is related to the integral of wall tension over time, the tension–time index [30, 31]. The tension–time index is fundamentally very similar to PVA if contraction time is approximately related to the theoretical change in volume of PVA. PVA leads to a clearer mechanistic understanding (pressure × volume has units of work while tension × time does not) and is, therefore, the focus here.

### Connection between pressure–volume relationships and ventricular function curves

**Fig. 6:**
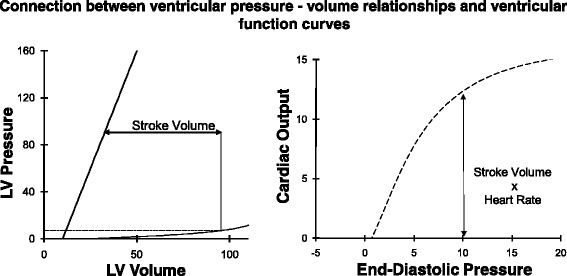
Stroke volume, derived from the *left-hand panel*, multiplied by heart rate yields cardiac output on the *right-hand panel*. Thus, for a given ESPVR, diastolic pressure–volume relationship, and afterload, a range of end-diastolic pressures on the *left-hand panel* yield the ventricular function curve on the *right-hand panel. LV* left ventricular

The pressure–volume loops of a cardiac cycle described above are directly related to ventricular function curves (Fig. 6). Stroke volume (determined as the difference between end-diastolic volume and end-systolic volume) multiplied by heart rate yields cardiac output. For a known systolic contractile state (ESPVR), diastolic pressure–volume relationship, and afterload, a unique stroke volume is determined for each end-diastolic pressure. Cardiac output can then be calculated yielding a ventricular function curve (Fig. 6). Since stroke volume is influenced separately by the ESPVR, the diastolic pressure–volume relationship, and afterload, it follows that ventricular function curves are affected by more than just changes in systolic contractility. For example, decreasing afterload results in increased systolic ejection, particularly in the failing heart. This results in increased stroke volume and cardiac output so that decreased afterload shifts the ventricular function curve up and to the left (Fig. 1). Conversely, decreased compliance (increased stiffness) of the diastolic left ventricle decreases stroke volume because end-diastolic volume is decreased at the same filling pressure. A decrease in contractility (ESPVR), decrease in end-diastolic compliance, or increase in afterload all decrease stroke volume and therefore shift the curve downward and cannot be distinguished on a cardiac function curve.

## Interaction with the vasculature

### Interaction of ventricular pressure–volume relationships with the arterial vasculature

**Fig. 7:**
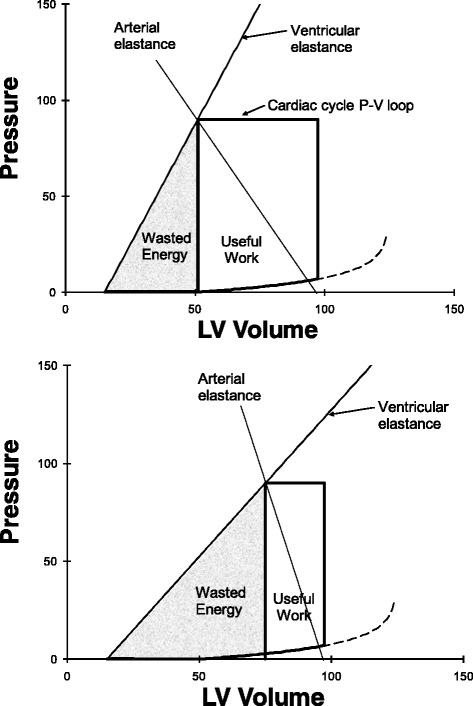
An arterial elastance line can be added to a PVA diagram (Fig. 5) by considering the rise in pressure within the arterial system with an increase in arterial volume (equals the decrease in ventricular volume). Compared with the healthy state (*top panel*), a decrease in ventricular elastance and an increase in arterial elastance mean that more energy is wasted on the PVA diagram. *LV* left ventricular, *P-V* pressure–volume

The left ventricle ejects blood into the aorta so left ventricular elastance interacts with aortic elastance during the ejection phase of systole when the aortic valve is open. Aortic elastance can be estimated from the slope of a plot of aortic pressure versus volume of blood ejected into the aorta (Fig. 7). An optimal ratio of stroke work (useful work) to PVA (wasted energy) of the left ventricle is achieved when left ventricular elastance is approximately equal to aortic elastance. To understand this consider two connected balloons. Note that elastance equals pressure change per volume change. If the balloons have equal elastance, then they are at equilibrium so the pressure × volume change in one balloon is exactly counterbalanced by the pressure × volume change in the other balloon; transferring volume from one balloon to the other can occur with no external work. Ventricular elastance greater than arterial elastance results in a pressure drop from the ventricular balloon to the aortic balloon, which results in lost energy [32]. If ventricular elastance is less than arterial elastance, then energy is wasted as potential mechanical work on a PVA diagram (Fig. 7).

In health left ventricular end-systolic elastance (end-systolic pressure divided by end-systolic volume) is similar but somewhat less than aortic elastance (slope of the aortic pressure–volume relationship, which is actually a “dynamic” elastance). Over the observed range stroke work and efficiency remain close to optimal [33]. During the ejection phase left ventricular elastance increases as described by Suga and Sagawa’s time-varying elastance. Interestingly, elastance of the aorta also increases during the ejection phase since the pliable aorta becomes stiffer as it fills during the ejection phase. Overall, there is reasonable matching of ventricular elastance to aortic elastance. This minimizes the amount of work done by the heart in transferring blood from the ventricle to the aorta. Energy due to blood volume stored in the elastant aorta is then available to drive blood flow through peripheral arteries during diastole. In a modeling study, Magder et al. [34] also included changes in resistance. They found that aortic compliance and the consequent elastance is only about one-eighth of ventricular end-systolic elastance. They surmised that changes in aortic elastance, as used by Burkhoff and Sagawa [32], may also reflect changes in resistance.

In disease states ventricular/aortic compliance becomes mismatched. Heart failure due to decreased systolic contractility means that ventricular systolic elastance in greatly decreased. If the cause is related to coronary artery disease, then it is very common to also find a diseased, stiff aorta. This accentuates the mismatch between ventricular and aortic compliance during systole, which means that a greater fraction of the work done by the ventricle is wasted (a greater fraction of PVA is under the ESPVR and is not stroke work; Fig. 7).

### Interaction of the heart with venous blood flow returning to the heart

The pressure–volume characteristics of the ventricle mean that if more blood returns to the heart (more diastolic filling so a greater V_ED_), then the ventricle will still eject to the same ESPVR so that stroke volume will increase exactly as much as V_ED_ increased. This gives the heart the basic characteristic that it will eject any amount of blood that flows back to it. This allows regulatory control of cardiac output to move to the peripheral circulation—a necessary feature because tissue hypoxia is best sensed by the peripheral vasculature where oxygen demand is occurring.

**Fig. 8:**
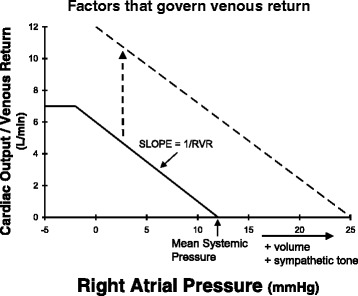
Lowering right atrial pressure increases blood flow back to the heart—venous return. This venous return curve can be shifted, primarily by an increase in the x-axis intercept—mean systemic pressure. *RVR* resistance to venous return

The factors governing venous return are illustrated in Fig. 8. When right atrial pressure equals mean systemic pressure there is no driving pressure gradient for blood flow back to the heart; thus, venous return is zero. As right atrial pressure is reduced the impediment to blood flow back to the heart decreases so that venous return rises approximately linearly. The slope of this venous return curve has units that are the inverse of a resistance—termed “resistance to venous return” [35]. When right atrial pressure is reduced to approximately zero the central veins collapse at the level of the diaphragm, limiting venous flow.

It can be seen that venous return at any right atrial pressure can be increased by increasing mean systemic pressure or by decreasing resistance to venous return. Mean systemic pressure is a vascular compartment pressure that arises from venous blood volume distending the systemic veins. If the volume of the distensible venous compartment is increased, then mean systemic pressure increases. Alternatively, if the capacitance of the venous compartment decreases, say by catecholamine infusion or increased sympathetic tone, then the pressure within the venous compartment can increase without a change in volume.

**Fig. 9:**
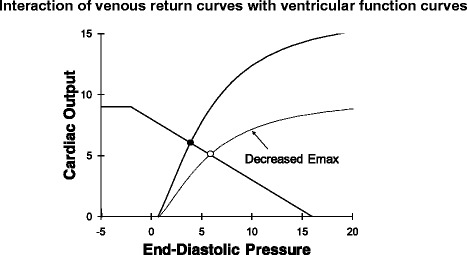
Ventricular function curves can be plotted on the same set of axes and venous return curves. In steady state cardiac output must equal venous return so the intersection (*filled circle*) identifies the cardiac output and end-diastolic pressure of the cardiovascular system. A decrease in cardiac function results in a decrease in cardiac output and an increase in end-diastolic pressure (*open circle*)

In steady state the amount of blood returning to the heart must equal the amount of blood leaving the heart so that venous return must equal cardiac output. Indeed, stroke volume must, on average, equal “stroke return”. Therefore, as illustrated in Fig. 9, the ventricular function curve and venous return curve can be plotted on the same set of axes. The intersection of the ventricular function curve and venous return curve give the value of steady state cardiac output and the value of steady state right atrial pressure.

## Implications for treatment of cardiac dysfunction

### Effect of inotropic agents and afterload on normal and failing heart function

**Fig. 10:**
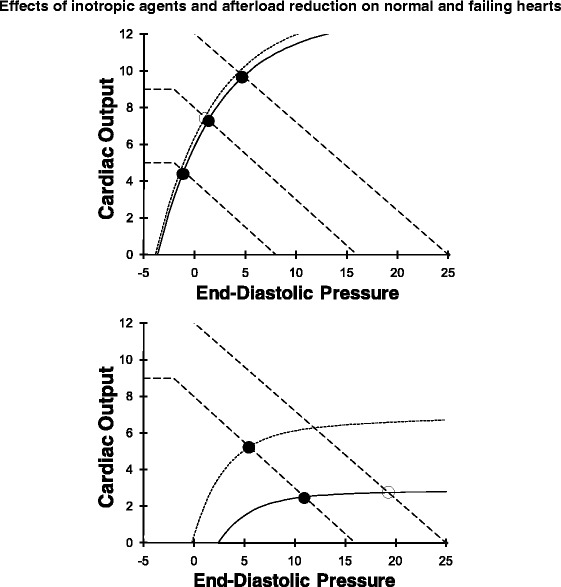
*Top*: in a normal heart increased contractility does not change the normally steep ventricular function curve so this does not change cardiac output much. In contrast, changes in venous return cause large changes in cardiac output so, in health, cardiac output is primarily controlled by the peripheral circulation. *Bottom*: in a failing heart changes in venous return curves no longer result in substantial changes in cardiac output but raise venous pressure (*solid circle* to *open circle* on bottom ventricular function curve). Now an increase in contractility results in a substantial increase in cardiac output and decrease in end-diastolic pressure (*filled circle* on lower ventricular function curve to *filled circle* on upper ventricular function curve)

In health, cardiac output is determined almost exclusively by the factors governing venous return (mean system pressure, resistance to venous return, right atrial pressure). The top panel in Fig. 10 shows that substantial changes in cardiac output occur with shifts in the venous return curve. In contrast, a shift of the ventricular function curve due to either an increase in ventricular contractility or a decrease in afterload hardly alters cardiac output because, in health, the ESPVR is already very steep (Fig. 10).

When Emax, the slope of the ESPVR, is low then doubling of Emax or a reduction in afterload leads to a very substantial shift up of the ventricular function curve. This results in a large increase in cardiac output at a decreased end-diastolic pressure and volume. Thus, increasing ventricular contractility is an ineffective strategy when the heart is normal but is a highly effective strategy to increase cardiac output when systolic contractility is reduced in a disease state (Fig. 10, bottom panel). Similarly, arterial vasodilators only reduce arterial pressure in healthy individuals but are very effective in increasing ventricular function and cardiac output when Emax is low.

## Conclusions

Many aspects of left ventricular function are explained by considering ventricular pressure–volume characteristics. Ventricular contractility, diastolic compliance, actual and potential stroke work, and the interaction of the heart with the arterial and venous circulations all can be elucidated from ventricular pressure–volume characteristics. Consideration of ventricular pressure–volume relationships highlight features that allow the heart to efficiently respond to any demand for cardiac output and oxygen delivery.

## Abbreviations

dP/dt_max_, maximum rate of change of ventricular pressure; Emax, maximum elastance; ESPVR, end-systolic pressure–volume relationship; PVA, pressure–volume area; SV, stroke volume; V_ED_, ventricular volume at end-diastole; V_ES_, ventricular volume at end-systole; Vmax, maximum velocity of shortening of the contractile element; ΔP/ΔV, elastance
